# A proof of concept reinforcement learning based tool for non parametric population pharmacokinetics workflow optimization

**DOI:** 10.1007/s10928-022-09829-5

**Published:** 2022-12-07

**Authors:** J. D. Otalvaro, W. M. Yamada, A. M. Hernandez, A. F. Zuluaga, R. Chen, M. N. Neely

**Affiliations:** 1 Laboratory of Applied Pharmacokinetics and Bioinformatics, Department of Infectious Diseases, Children’s Hospital Los Angeles, Los Angeles, CA USA; 2 Bioinstrumentation and Clinical Engineering Research Group, Engineering Department, University of Antioquia, Medellín, Colombia; 3 Laboratory of Integrated and Specialized Medicine, Medical School, University of Antioquia, Medellín, Colombia

**Keywords:** Pharmacokinetics, Reinforcement learning, Automation, Modeling process, Modeling

## Abstract

The building of population pharmacokinetic models can be described as an iterative process in which given a model and a dataset, the pharmacometrician introduces some changes to the model specification, then perform an evaluation and based on the predictions obtained performs further optimization. This process (perform an action, witness a result, optimize your knowledge) is a perfect scenario for the implementation of Reinforcement Learning algorithms. In this paper we present the conceptual background and a implementation of one of those algorithms aiming to show pharmacometricians how to automate (to a certain point) the iterative model building process.We present the selected discretization for the action and the state space. SARSA (State-Action-Reward-State-Action) was selected as the RL algorithm to use, configured with a window of 1000 episodes with and a limit of 30 actions per episode. SARSA was configured to control an interface to the Non-Parametric Optimal Design algorithm, that was actually performing the parameter optimization.The Reinforcement Learning (RL) based agent managed to obtain the same likelihood and number of support points, with a distribution similar to the reported in the original paper. The total amount of time used by the train the agent was 5.5 h although we think this time can be further improved. It is possible to automatically find the structural model that maximizes the final likelihood for an specific pharmacokinetic dataset by using RL algorithm. The framework provided could allow the integration of even more actions i.e: add/remove covariates, non-linear compartments or the execution of secondary analysis. Many limitations were found while performing this study but we hope to address them all in future studies.

## Introduction

Population pharmacokinetic (pop-PK) modeling is a fundamental task in drug development and use. The culmination of the task is to choose the most informative or trusted model from a set of best-fit models on the dataset. Finding a set of best-fit models is typically done via trial and error, guided by both the experience obtained modeling similar drugs and the most recent trials of the current modeling task. Although the chosen model must satisfy both quantitative and qualitative measures of goodness of fit, the process of fitting any model to the dataset is purely quantitative. This work explores using only quantitative measures to develop a small set of best-fit models for pharmacometric consideration.

In non-parametric pop-PK no assumption is made regarding the family of statistical distributions from which model parameters are drawn; thus, the pop-PK modeling task requires searching for both best-fit model structure and best-fit model parameter ranges.

Optimization of Pop-PK model often relies on a trial-and-error phase in which the modeler makes a change on the model, fits it again and then evaluate the results obtained and decide if further changes need to be made. By restricting the decision making during this trial-and-error phase to only quantitative elements we intend to allow the pop-PK task to be rewritten as a reinforcement learning algorithm.

More than presenting a solution, this article seeks to conceptually introduce pharmacometrists of all training levels to the implementation of their own solutions using this type of algorithm. This is the reason why we expand on the presentation of the concepts related to the construction of the reinforcement learning algorithm, we want this article to also serve as an introduction to the topic for pharmacometrists who are not yet involved with machine learning.

Here, we report the initial implementation of an autonomous algorithm using Reinforcement Learning that can guide or independently perform sections of the non-parametric pop-PK model selection process. Providing a tool that relieves the modeler of some of the burden of model selection, particularly early in the iterative process, allows more efficient use of time to promote and leverage the pharmacometrician’s creativity and insight during the pop-PK task.

## Theoretical

Pharmacometricians execute a sequential design process. Each step has three conceptual parts: the data is compared to the current model predictions and a hypothesis is formed, a model is constructed, and an optimization performed. This is repeated until comparison of data and model prediction is satisfactory. The iterations of this sequence are an informed exploration of potential models. In this work, we remove hypothesis formation (which requires human intelligence) and determine new models at each iteration step based solely on optimization performance of the current model (and without human intervention).

Since optimization performance is one of many potential metrics of how informative a model is on the data, the need for human intelligence is not alleviated, but the initial (and usually most time-consuming part of the pharmacometrician’s task) is automated: that is, given a small set of parameterized models, find the best fit on the data.

The task of determining, from a set of ”best-fit” models, which is the most informative to the specific question asked of the modeler is one that cannot be answered solely through automated design because unlike the question of best fit, which has a true metric, the question of most informative also relies on qualitative assessment, in the case of maximum likelihood methods this metric is the likelihood or one of its transformations (log-likelihood, Akaike Information Criterion, Bayesian Information Criterion,...).

### Pharmacokinetic models

Pharmacokinetics (PK) is the study of the absorption, distribution, metabolism, and elimination processes of a drug in the body and the mathematical equations to model these processes. In pop-PK, the usual way to represent these systems is by compartmental models. A compartmental model is a mathematical construction in which the body is represented as a set of ”interconnected tanks”. Drug flows between the tanks and is assumed to be instantly and evenly distributed within each of these tanks.

Tanks which correspond to measured drug concentrations are assigned a volume $v_i$ to relate the amount of drug inside it $X_i$ to the concentration. The connections that dictate drug mass transfer between the environment and tanks and between tanks are typically represented by a rate $k_{ij}$ , where i is the originating tank and j is the destination, and 0 can be used to represent the environment. An alternative parameterization of mass transfer is clearance terms describing drug-containing volumes, e.g., $Cl_{ij}$ or $Q_{ij}$.

Simple PK models without pharmacodynamic (PD) outputs usually contain one to three compartments [14], depending on the need to describe absorption from extravascular drug administration (e.g. oral) and/or linear or non-linear decline of log-transformed drug concentrations with respect to time after dosing. It is important to highlight that pop-PK modeling is usually empiric in that equations are derived to mathematically fit the shape of the concentration-time profiles without regard to underlying mechanistic reasons for the shapes of the profiles. While understanding of anatomy or physiology can inform choice of compartments and types of transfers, detailed mathematical equations with large numbers of parameters to describe such processes are characteristic of a physiologically based PK (PBPK) approach and not a pop-PK approach [13]. Little physiology is associated with non-PBPK models, except for the systemic concentration in the central compartment [6].

Equations (1) and (2) are the analytic solution for one and two compartments models respectively, adapted from [4]:

1 $$\begin{aligned}&X(t)=X(0)e^{-k_e.t}+\frac{R}{k_e}(1-e^{-k_e.t}) \end{aligned}$$

2 $$\begin{aligned}X_c(t)=&\frac{X_c(0)}{\lambda _1 + \lambda _2} ((\lambda _1 -k_{pc})e^{-\lambda _1.t} \nonumber \\&- (k_{pc}-\lambda _2)e^{-\lambda _2.t}) + \frac{R}{\lambda _1 + \lambda _2}\left( \frac{(\lambda _1 -k_{pc})}{\lambda _1}(1-e^{-\lambda _1.t})\right. \nonumber \\&\left.- \frac{(k_{pc}-\lambda _2)}{\lambda _2}(1-e^{-\lambda _2.t})\right) \end{aligned}$$

with

3 $$\begin{aligned}&\lambda _1,\lambda _2 = \nonumber \\&\quad \frac{(k_e + k_cp + k_pc) \pm \sqrt{(k_e + k_cp + k_pc)^2-4k_ek_pc}}{2} \end{aligned}$$

### Non-parametric maximum likelihood methods

Our laboratory has long favored non-parametric (NP) pop-PK methods. The mathematical requirements for applying likelihood as a guiding metric for NP-PK model development were first completely expressed by [10]. Mallet’s paper relates likelihood and optimal design theory in the context of PK. One conclusion is the maximum likelihood (ML) solution is a number of discrete, weighted support points less than or equal to the number of subjects.

Thus, algorithms that rely on likelihood to guide model development maximize equation

4 $$\begin{aligned} L( w, \phi ) =\prod _{i=1}^N \sum _{k=1}^K w_k p( Y_i \vert \phi _k ) \end{aligned}$$

with respect to the support points ${\phi } = ({\phi }_1, \ldots , {\phi }_K)$ and weights ${w} = ( w_1, \ldots , w_K)$ such that ${\phi }_k \in \Theta , w_k \ge 0$ for $k=1, \ldots , K$, $K \le N$ and $\sum _{k=1}^K w_k = 1$. Bender’s decomposition is often invoked to allow the above optimization to be carried out in two parts, optimization of support point placement and optimization of their weights [2]. The distribution that maximizes equation (4) is a consistent estimator of the true mixing distribution; which means that it will converge to the true distribution if the number of subjects is large. This was proved by [7].

By construction, NP methods converge to a local optimal solution given the search space boundary, initial estimated support point locations and the error function of the associated regression model. Over 20 years ago, LAPKB developed the Non-parametric Adaptive Grid (NPAG) algorithm [16] and recently the faster Non-parametric Optimal Design (NPOD) algorithm [8].

In this work, NPOD is initialized using a Sobol sequence of 51 elements within the input boundary, and the error is fixed to be constant. An interior point method (IPM) is used to find the weights of each point such that the likelihood is maximized. The support points with high probability are saved as the initial support.

#### NPAG

NPAG is a iterative grid search algorithm, that uses a grid expansion/contraction add extra support points to the pool $\{ \phi _k \}$, then, by determining $p( Y_i \vert \{ \phi _k + \phi _{extra} \})$, applies a primal-dual interior point algorithm to choose the best *N*-or-less supports from this pool, consistent with equation (4); $\{ \phi _k + \phi _{extra} \} \rightarrow \{ \phi _{k+1} \}$. The algorithm ends when no improvement can be found. NPAG was first introduced by Leary [8] and details of the algorithm are found in [16]. NPAG and NPOD differ in their method for determining the extra support points given the current support.

#### NPOD

The initial set of support points are saved as an ”anchor” set. Each support point is allowed to move in turn, leaving all other points in the anchor at their position. Usually a Nelder-Mead algorithm is applied to move the points, and likelihood is used as the objective during movement. The resulting ”optimal” points are now added to the anchor set, the IPM is applied, and the less likely points are removed. This procedure is repeated to convergence. Details about the NPOD algorithm are about to be published elsewhere, but the general idea is presented in this poster by Leary et al. [8].

### Machine learning tools

#### Markov decision process

Markov Decision Process (MDP) is a framework to define decision-reward based problems. The decision maker is called *agent*, this agent interacts with the *environment*, changing the environment’s state and receiving a reward. The MDP framework proposes than any goal-directed problem can be reduced to three elements that are being interchanged between an agent and an environment: the choice made by the agent *action*, the environment’s *state* and the *reward* that indicates the agent if it’s reaching its goal [15]. The sequence of actions is taken until receiving a termination signal is called an *episode*.

The agent interacts with the environment in discrete time steps, on each step the agent has access to pool of actions $a \in A$ and can select one to perform. After this the environment gives the agent a new observation and a reward associated $r \in R \subset {\mathbb {R}}$. An observation is a (partial) representation of the environment’s state $s \in S$. A MDP can be defined by the quintuple $M=\langle S,A,P,R,\rangle$ [9] where P are the transition probabilities and $\gamma$ is the discount factor, these concepts are going to be defined later in this document.

**Fig. 1:**
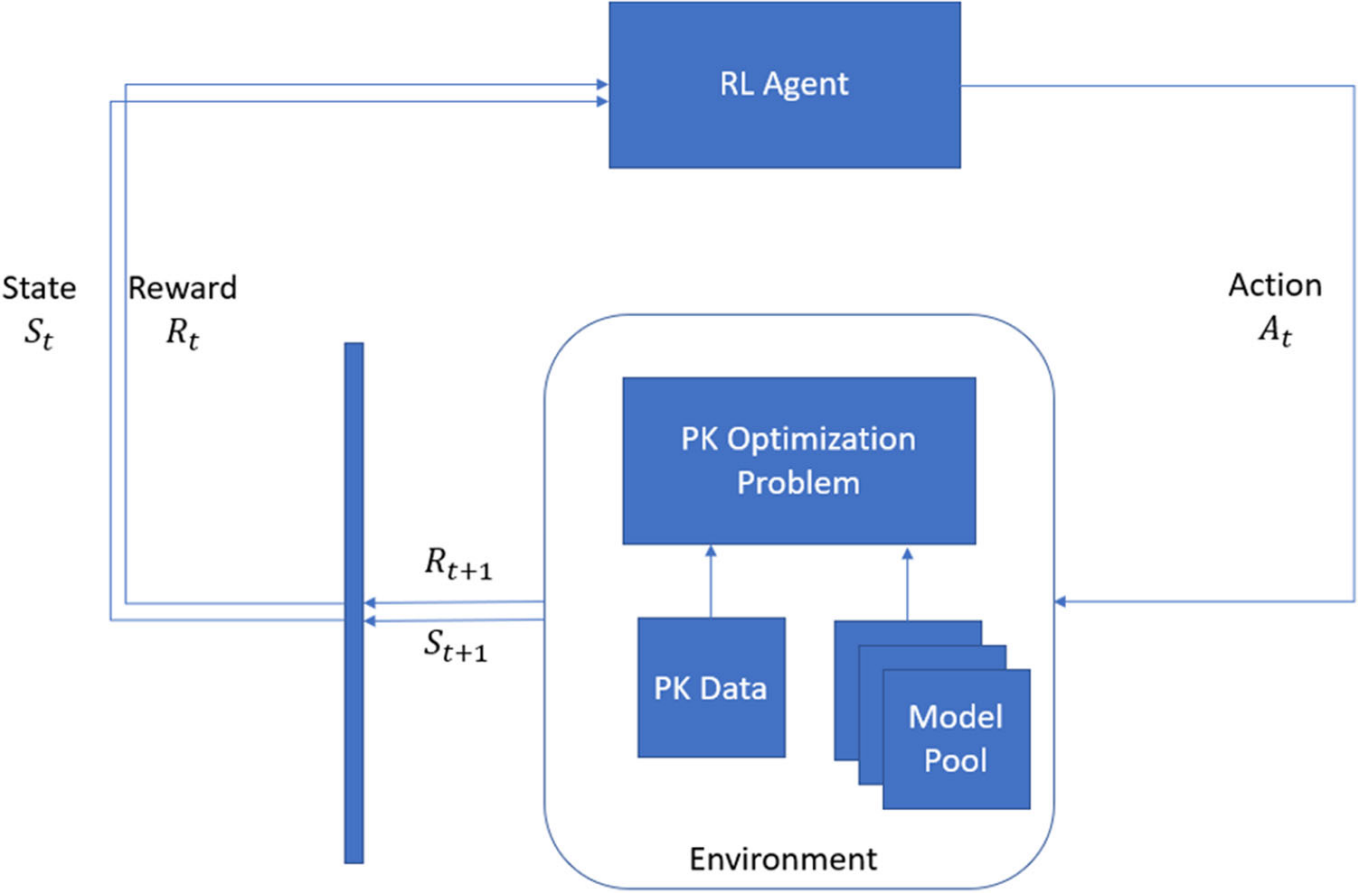
PK Optimization problems in terms of RL. *RL* Reinforcement learning, *Pk* Pharmacokinetic, *St* State at time t, *Rt* Reward at time t, *At* Action at time t

In the pharmacokinetic modeling case the agent would be the pharmacometrician, the environment is the software where he is modeling his PK data with, the actions would be the decisions he makes to fit the model and the reward would be a quantity he uses to evaluate the fitness of his model. The termination signal would be the moment when the pharmacometrician realizes that his criteria is met or that the model is good enough an decides to stop the modeling process. Figure 1 represents the PK optimization problem defined by a dataset and a pool of models in terms of the RL framework.

#### Reinforcement learning

Reinforcement Learning (RL) is one of the three main branches of machine learning. It is defined by a set of tools that aim to train different kind of agents over an MDP in how to take decisions to maximize the total expected reward on a defined time horizon.

A critical problem that needs to be addressed in RL is the trade-off between exploration and exploitation. In an ideal scenario an agent would prefer to *exploit* actions that have proven to return a high reward, but in order to obtain that knowledge the agent has to *explore* them or similar actions before . In their introduction to RL [15] expresses that the equilibrium between exploration an exploitation is an open problem that for example does not need to be addressed in supervised or unsupervised learning.

This way of learning is very reminiscent of the way living beings learn. Taking actions, analyzing the consequences and internalizing the results to make better decisions in the future, while taking into account the balance between making safe decisions to get an ensured reward or when to explore new options. And because these RL-driven agents does not have the time, computational capacity or memory constraints humans do, they will end up evaluating sequences of actions that might not be obvious to a modeler from the very beginning.

#### Other RL concepts

- The future reward $G_t$ also known as return is the total sum of discounted rewards in an infinite time horizon $G_t=R_{(t+1)}+\gamma *R_{(t+2)}+ \cdots =\sum _{k=0}^{\infty } \gamma ^k*R_{(t+k+1)}$.
- The discount factor $\gamma \in [0,1]$ describes how much the algorithm is going to value future rewards versus present ones, the closest to 1 the most importance the algorithm is going to give to future rewards.
- Learning rate $\alpha$ is a hyper parameter that defines how fast we want the agent to learn, a learning rate of 0 means that the agent is not going to learn any new information and a learning rate of 1 means that the agent is going to prioritize the new information over the old.
- The agent’s policy $\pi (s)$, that describes the best action to take in a given state to maximize this total sum of rewards. This policy can be deterministic $\pi (s) = a$ or stochastic $\pi (a|s) = P_\pi [A=a|S=s]$.
- The value of an state $V_\pi (s)$ is defined as the total sum of rewards the agent will get if it is in the state s and then follows the policy $\pi$ until termination. $V_\pi (s)={\mathbb {E}}_\pi [G_t|S_t=s]$.
- The value of an state-action pair $Q_\pi (s,a)$ similarly to $V_\pi (s)$ is the total sum of rewards the agent might obtain if it is in the state s, but in this case it is going to take action a and then follow the policy $\pi$. $Q_\pi (s,a)={\mathbb {E}}_\pi [G_t|S_t=s,A_t=a]$.
- On-policy and off-policy distinguish between to different kinds of RL algorithms, in the former the agents is trained by using trajectories that follow the agent’s policy, in the latter the agent learns a different policy that the one it is actually following in the training process.
- Model-based RL is the subset of algorithms the transition probabilities P and rewards R are known beforehand. And finally, model-free RL defines the algorithms in which the learning process is independent of P and R. The latter is the case in the pharmacokinetic model building process. Have in mind that P and R are two of the 5 elements that defines the MDP problem.

The end goal of RL is to train the agent into maximize the total sum of discounted rewards, for finite MDPs this means finding the optimal policy $\pi ^*$. This policy is defined as $v_{\pi ^*}(s) \ge v_\pi (s)$ for all $s \in S$. In the same way the state-action pair for an optimal policy can be expressed as follows, adapted from [15]:

5 $$\begin{aligned} Q_{\pi ^*}(s,a) = {\mathbb {E}}[R_{t+1}+\gamma v_{\pi ^*}(S_{t+1})|S_t=s,A_t=a] \end{aligned}$$

Because of this relationships, there are two different approach to maximize this total sum of discounted rewards. In value learning methods the goal is to train the agent to learn $v_\pi (s)$ or $Q_\pi (s,a)$. Or in policy learning methods when the agent looks to infer $\pi$ directly.

#### Dynamic programming

Dynamic Programming (DP) refers to the set of algorithms used to solve optimization problems by dividing it up into multiple sub-problems each of them can be optimized independently by computing an optimal policy. This is what Bellman calls the ’principle of optimality’, in his words: ”an optimal policy has the property that, whatever the initial state and initial decisions are, the remaining decisions must constitute an optimal policy with regard to the state resulting from the first decision” [1].

The central idea of DP is to use value functions to organize the search of better policies, this is done in three steps: policy evaluation, policy improvement and policy iteration to find a $v_\pi (s)$ or $Q_\pi (s,a)$ that satisfies the Bellman’s optimality Eq. (6), for the value version of this equation and a more in deep explanation of these topics refer to [15]

6 $$\begin{aligned} Q_{\pi ^*} = \sum _{s',r}p(s',r|s,a)[r + \gamma \underset{a'}{max}Q_{\pi ^*}(s',a') ] \end{aligned}$$

One property of interest of DP methods is that they update their estimates of the value-action pairs using the values of their successor as is evident in (6), this idea is called bootstrapping. By using this concept RL algorithms are able to backward propagate the information about good state-action pairs to the beginning of the episode.

#### Monte Carlo methods

Monte Carlo (MC) methods in RL are used to estimate the value functions and by this discovering optimal policies, unlike DP algorithms MC methods does not need to know the transition probabilities or reward accurately beforehand. Monte Carlo methods relies on the *exploration* of a real or virtual environment, acquiring experience by sampling sequences of actions, states and rewards on multiple episodes. A difference between Monte Carlo methods with DP is that the former only adjust rewards at the end of each episode, this imposes a set of restrictions: each episode must terminate no matter the sequence of actions taken and they average complete returns, it is not possible to learn from partial results and they not bootstrap.

A benefit MC methods offer over DP that is specially relevant in the population pharmacokinetic model selection process is the fact that they can learn how to behave optimally just by analyzing these exploration steps over the environment, in other words, it would be possible to capture the state-action-sequence taken by an expert and introduce that knowledge to the agent directly.

To guarantee convergence in this random exploring process, MC methods implements greedy policy, that is the one that for each $s \in S$ chooses the action that has the maximal action-value, theorem presented in (7) is borrowed from [15] and shows how by having a greedy policy the algorithm can guarantee that in a given step *k* $\pi _{k+1}$ is going to be as good or better than $\pi _k$.

7 $$\begin{aligned} \begin{aligned} \pi (s)&\,{\dot{=}}\, \underset{a}{arg max} Q(s,a)\\ Q_{\pi k}(s,\pi _{k+1}(s))&= Q_{\pi k}(s, \underset{a}{arg max} Q_{\pi k}(s,a))\\&= \underset{a}{max} Q_{\pi k}(s,a)\\&\ge Q_{\pi k}(s, \pi _k(s))\\&\ge v_{\pi k}(s). \end{aligned} \end{aligned}$$

The main problem is the fact that the agent needs to explore and take random actions in order to acquire experience but it also needs to take greedy actions to be able to converge, obtaining equilibrium between these two divergent behaviors is a key aspect of Reinforcement Learning algorithms. One way to do it is by using $\epsilon$-greedy policies, this mean that that the agent is going to balance exploration and exploitation by taking a greedy action with a probability $1 - \epsilon + \frac{\epsilon }{|A(s)|}$ and all non greedy actions with a probability $\frac{\epsilon }{|A(s)|}$ each one.

#### Temporal difference reinforcement learning

Temporal Difference (TD) learning is the combination of Monte Carlo and Dynamic Programming ideas. TD can learn from raw experience like MC and can also bootstrap the information like DP. A key difference between MC and TD methods is that the latter does not need to wait until the end of the episode to update $Q_\pi (s,a)$, as they update the state-action pair after each time step.

In summary, the main characteristics of TD algorithms are:

- They do not require previous knowledge of the model (model free).
- They can be implemented in an incremental way.
- They do not need to wait until the end of the episode to learn.
- The exploration is not penalized as bad as other algorithms.

#### SARSA

SARSA (State-Action-Reward-State-Action) is an on-policy TD algorithm, as in other on-policy algorithms its goal is to estimate the value matrix $Q_\pi (s,a)$ for all states s and actions a, following an $\epsilon$-greedy policy $\pi$. This is done by taking the quintuple $(s_t, a_t, r_{t+1}, s_{t+1}, a_{t+1})$ and updating $Q_\pi (s,a)$ after every transition from $s_t$ to $s_{t+1}$ using Eq. (8) a modified version of Bellman’s equation .

8 $$\begin{aligned}Q(s_t,a_t) =& Q(s_t, a_t) + \alpha (r_{t+1} \nonumber \\&+ \gamma Q(s_{t+1}, a_{t+1}) - Q(s_t, a_t)) \end{aligned}$$

The general idea of the SARSA algorithm is to bootstrap future information our policy $\pi$ could provide while evaluating present rewards, how relevant each of those elements is going to be, is controlled by the parameters $\gamma$ and $\alpha$ respectively. The final algorithm used to train the agent in this paper is an interpretation made by Sutton and Barto [15] of the original work by G. Rummery and M. Niranjan [12] and presented in Algorithm 1

Because the final goal of SARSA is to estimate $Q_\pi (s,a)$, this imposes some limitations, in this case the most relevant is that both the state space S and the action space A should be finite and discrete. This is because the conditions of convergence Monte-Carlo methods imposes and the general assumptions of the algorithm. We explain our approach to overcome those limitations.
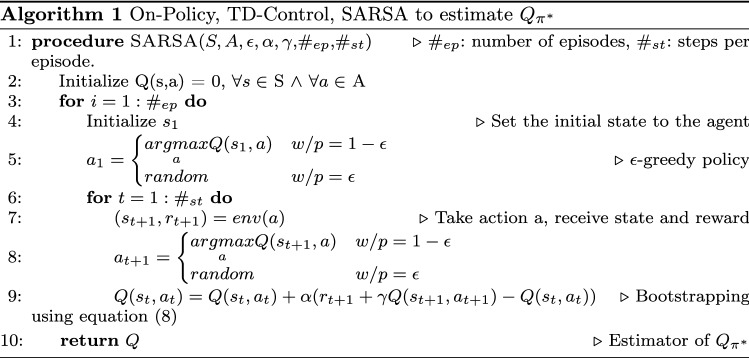


## Methods

### Data and models

We analyzed the behavior of the SARSA using a previously published dataset. In this scenario, the simulated population was initially described by [11] and modeled using NPAG. The dataset was of interest because it included a combination of unimodality, bimodality and outliers to test the RL agent and also because it is a simulated dataset, we knew the original distribution. For this exercise we restricted the structural models to one and two compartments, i.e., Eqs. (1) and (2). The coefficients of the error polynomial were fixed to the original values. No covariates were included in this analysis.

### Action space

The regular search space for a parameter is defined by its lower and upper limit. The upper and lower limit for each of the limits could be increased or decreased. This means that the agent would have four actions per parameter plus one action to switch between models. This means that when the agent is trying to fit (1) with two parameters, it would have 9 actions, but if the agent is trying to fit (2) with four parameters, it would have 17 actions. Lists (3.2) and (3.2) enumerate both sets of actions.

1. Increase lower bound of $k_e$.
2. Increase upper bound of $k_e$.
3. Increase lower bound of *v*.
4. Increase upper bound of *v*.
5. Decrease lower bound of $k_e$.
6. Decrease upper bound of $k_e$.
7. Decrease lower bound of *v*.
8. Decrease upper bound of *v*.
9. Switch to 2 compartments.

**List 1** Available actions for a one compartment model

To overcome the limitation of a discrete action space SARSA imposes, we decided to define discrete increase step size to $\frac{1}{10}=10\%$ and in order to guarantee consistency between opposing actions and by this making finite the action-state space, the decrease step size was fixed to $\frac{1}{11}\approx 9.1\%$.

1. Increase lower bound of $k_e$.
2. Increase upper bound of $k_e$.
3. Increase lower bound of *v*.
4. Increase upper bound of *v*.
5. Decrease lower bound of $k_e$.
6. Decrease upper bound of $k_e$.
7. Decrease lower bound of *v*.
8. Decrease upper bound of *v*.
9. Increase lower bound of $k_{cp}$.
10. Increase upper bound of $k_{cp}$.
11. Increase lower bound of $k_{pc}$.
12. Increase upper bound of $k_{pc}$.
13. Decrease lower bound of $k_{cp}$.
14. Decrease upper bound of $k_{cp}$.
15. Decrease lower bound of $k_{pc}$.
16. Decrease upper bound of $k_{pc}$.
17. Switch to 1 compartment.

**List 2** Available actions for a two compartments model

### State definition

Given the limitation of having a discrete state, the actual parameters ranges values does not really matter, we just need a key to differentiate different states based on the current value of the ranges and the number of compartments used.

The strings defined in 9, describe the state definition in the one and two compartments scenarios used to index $Q_{\pi }(s, a)$. $\uparrow$ and $\downarrow$ represent the upper and lower bound respectively.

9 $$\begin{aligned} \begin{aligned}&''1:\{\downarrow k_e , \downarrow v , \uparrow k_e , \uparrow v\}''\\&''2:\{\downarrow k_e , \downarrow v , \downarrow k_{cp} , \downarrow k_{pc} , \uparrow k_e , \uparrow v, \uparrow k_{cp} , \uparrow k_{pc}\}'' \end{aligned} \end{aligned}$$

### Reward

The reward was defined as follows, each Maximum Likelihood method return a log-likelihood at the end of each run, for the first run in each episode the reward is going to be equal to the likelihood achieved by taking the first action. Consequent rewards will be calculated as the difference between the current obtained likelihood and the previous one $\Delta _{LL}$. This was done this way to prevent the total sum of discounted rewards to be dependant on the number of steps in each episode.

We defined our reward function this way because of another implementation detail. Given the nature of this this reward definition, any improvement over the last reward obtained is going to be coded as a positive reward, the opposite is also true with negative rewards. Because we defined the initialization $Q(s,a) = 0$, each time the $\epsilon$-greedy policy selects to take a greedy action in a poorly explored state, there is going to be a preference to take non-previously explored actions (with reward 0) over previously non-effective actions taken (with a negative reward).

### RL parameter configuration

SARSA was configured using the following parameters: $\alpha = 0.9$, $\gamma = 1$, $\epsilon = 1/episode$, $\#_{episodes} = 1000$, and a time horizon of 30 actions per episode, thus a theoretical maximum total of 30.000 calls to NPOD. The initial state for the environment is: 2 compartments model with $k_e =[0.001,2]$, v=[125, 625], $k_{cp}=[0.001,10]$, $k_{pc}=[10,100]$ and an initial reward of 0. That initial condition is on purpose deviated of the real position of the simulated subject’s real parameter values: $k_e =[0,1]$, v=[50, 200] using one compartment model.

### Evaluation of performance

The results obtained will be evaluated in multiple ways, being the most important one the final likelihood obtained (giving that this is the way the reward was encoded). Even so the final number of support points, the shape of the distribution and more technical elements like the total amount of time spent in the training will be taken into consideration.

## Results

### Implementation

The final implementation is composed of different pieces in different programming languages, we are using the fortran implementation for the interior dual point method [16] included in the Pmetrics R package [11]. That code was compiled into a shared library used by our new implementations of the NPOD algorithm using the julia programming language [3].

**Fig. 2:**
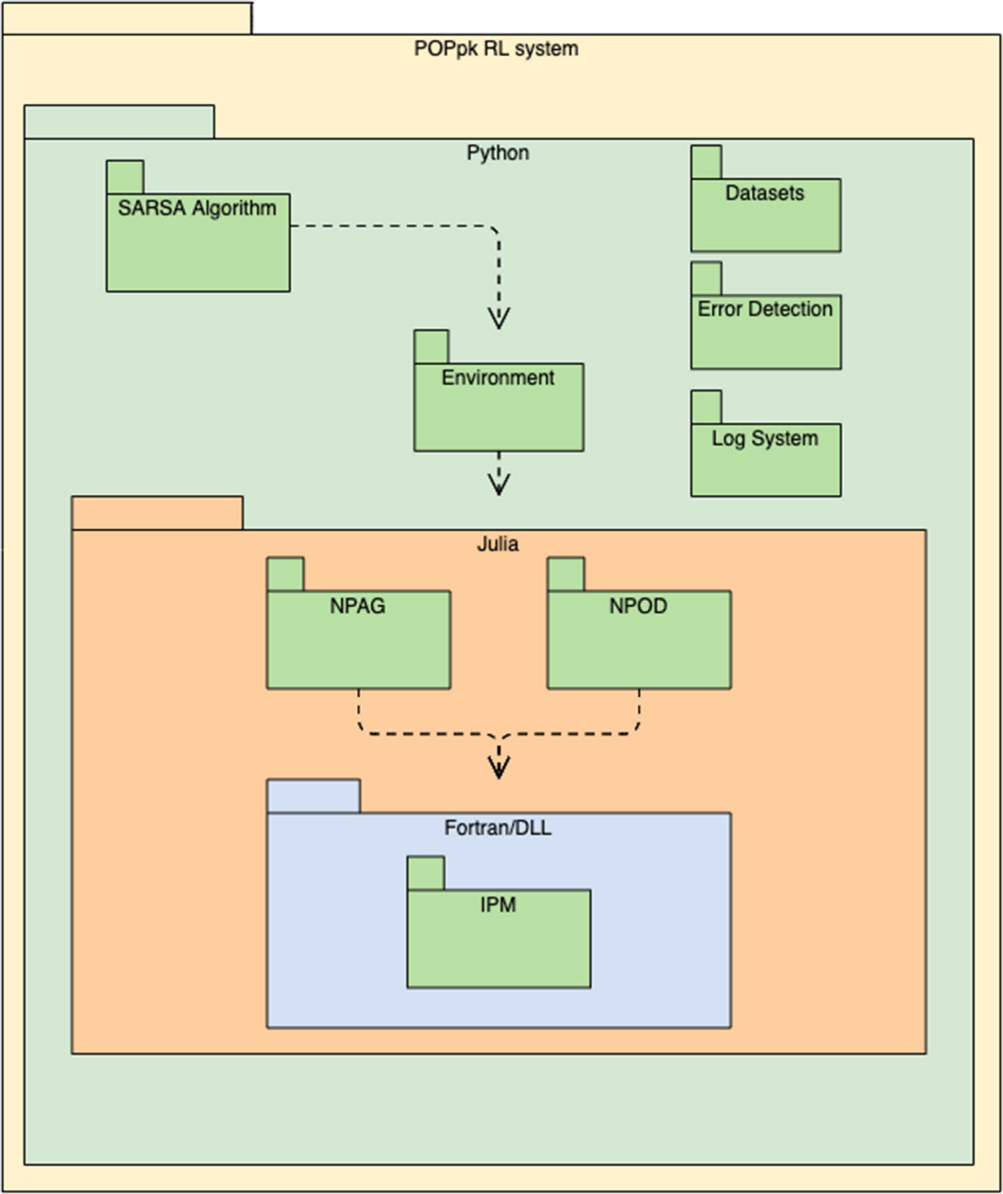
Package diagram of the RL implementation. Dotted arrows notate package dependencies. Requirements for a Functional PopPk RL System

The NPOD package was imported from julia into python to be used in the environment class, this class contains all the logic to manage the actions, state and observations generations and reward calculation when taking the actions. Finally, SARSA was implemented in python altogether with some other utilities to keep track on the changes of the reward, execution times and error catching. A package diagram of the whole implementation is presented in Fig. 2

### Learning

**Fig. 3:**
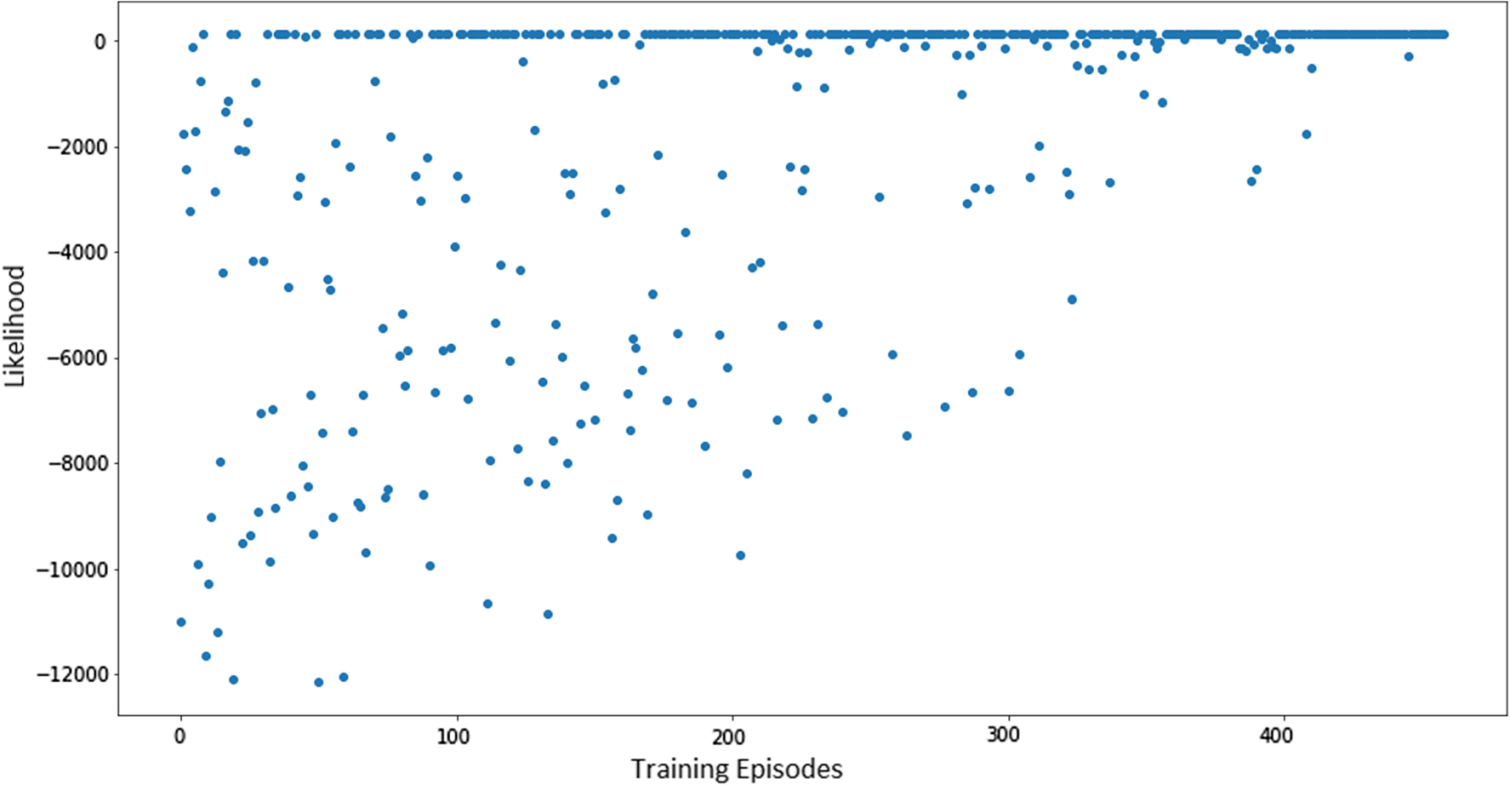
Reward obtained over 1000 episodes using NPOD as the driver of the optimization, aborted episodes were discarded

The total sum of rewards over each episode is presented on the Fig. 3, it is important to clarify that each step of an episode is a full run of the proper algorithm it is working with. It is clear how this total sum of rewards is increasing over time as the agents were collecting experience and started taking greedier actions.

Although the program was parameterized to perform 30 steps per episode, this might not be always the case, if in its exploration phase the agent takes actions in which the search space doesn’t contain a good density of support points the programs could basically return an error. In this case the reward would be hardcoded as -20.000 and the episode would end.

This is the reason in both figures we see less than 1000 episodes in the x-axis. We found that this behaviour particularly common when the agent switched from one to two compartments, given that the increase in the dimensionality of the space would reduce dramatically the density of support points. For the NPOD driven RL agent, a total of 55% of all the episodes were aborted.

### Evaluation

The total execution time to finish the 1000 episodes with 30 actions per episode was 5.5 h, using a machine with a AMD Ryzen 5950x.

**Fig. 4:**
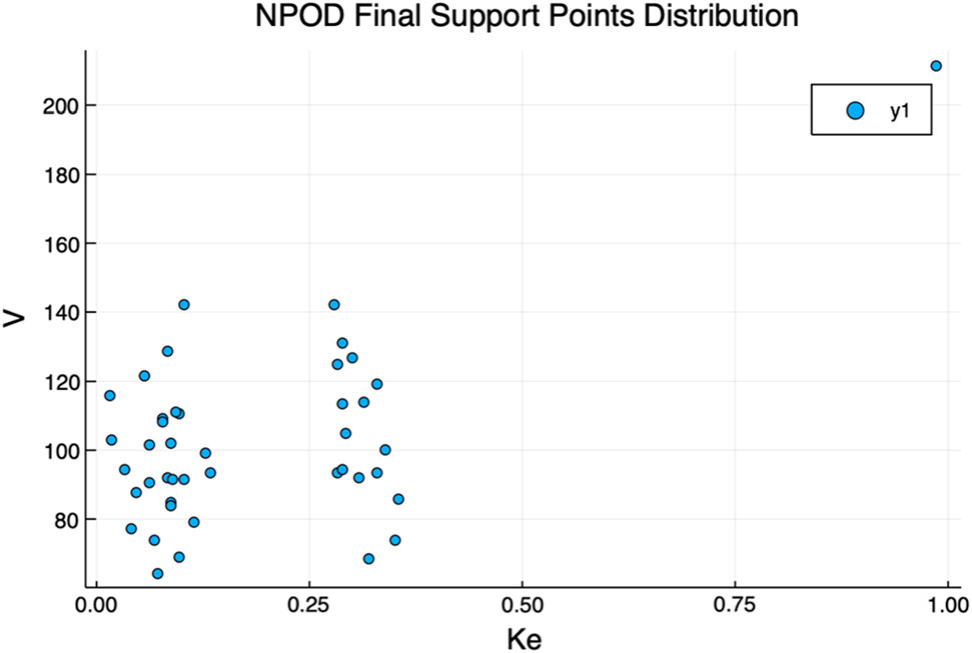
Ke and V values for the support points obtained automatically by the RL agent

At the end of those episodes, the final reward obtained by the agent in its learning process was equal to the obtained by the pharmacometrician to the second decimal digit. The optimization routine obtained 45 final support point and selected a model with two compartments over the one with only one. The distribution of $k_e$ and V obtained by the RL agent is shown in Fig. 4. For reference 5 shows the same plot for the original simulated subjects and the support points obtained in the original paper [11].

**Fig. 5:**
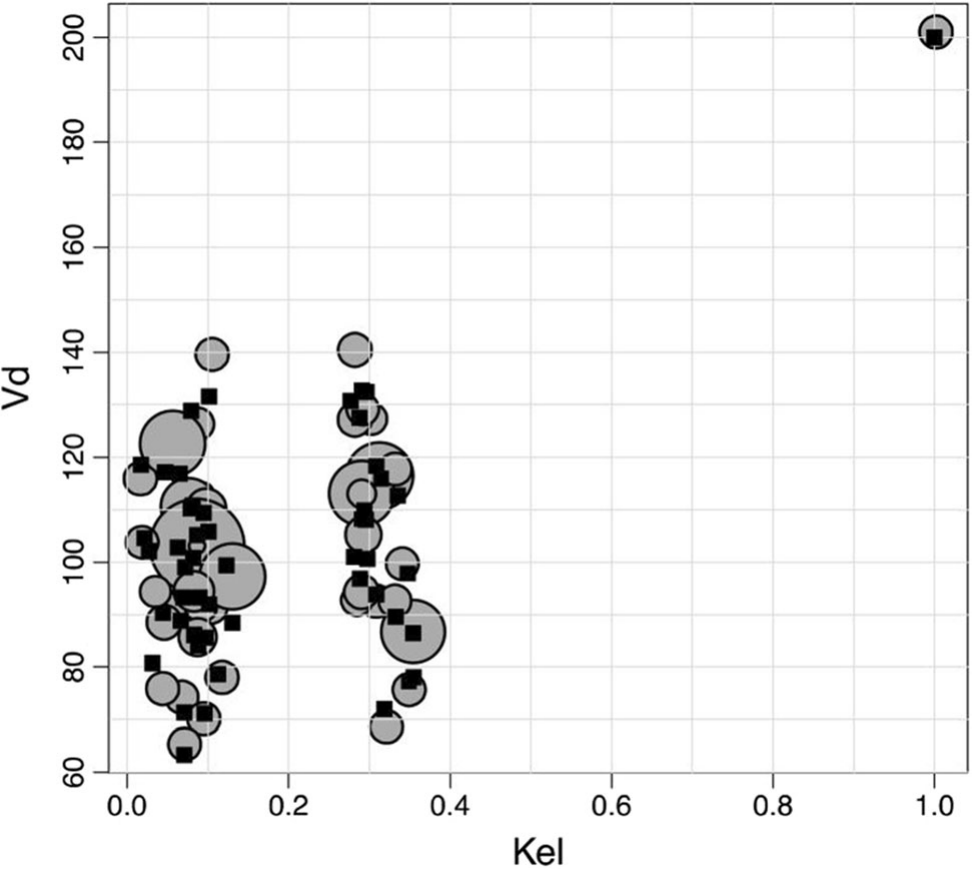
Distribution of ke and V. The black squares represent the real position of the simulated population while the gray circles represent the result obtained by a pharmacometrician the radius of the circle is proportional to the probability of each support point. Image courtesy of [11]

## Discussion

### Limitations

Theoretically, we could use an infinite amount of time and computational power to memorize $Q_\pi (s,a)$ for all state-action pairs, but this approach rapidly becomes computationally infeasible whit larger action spaces. Thus, there are techniques to approximate Q values using Neural Networks, they can generalize previous acquired knowledge into unexplored state-action spaces.

Likelihood was chosen as a measurement of reward because it is a straightforward implementation, but it also comes with its own set of downsides, for example: It would not support changes in the error polynomial coefficients or certain changes in the structural model, it also does not consider another set of external values like execution time or computational resources used.

Given the limitations of SARSA, here we are using discrete state and actions representations. This does not align with reality in the sense that modelers could switch from one to three compartments, while varying the parameter ranges by some amount in only one step.

We think that the main limitation this kind of implementations have is the amount of time needed to train the agent, especially because of the need to run the full optimization for each step in each episode. We expect that a distributed implementation, a reduction in the requirements of convergence for the Maximum Likelihood methods or using a Deep Reinforcement Learning technique or a combination of them could be good candidates to overcome this limitation.

In this paper we present an implementation of a known RL algorithm as a proof of concept of the utilization of these concepts in the automation of non-Parametric model building. And although is not the first time RL is used in the field, no further advancement have been done since 2013 [5]. As stated before, the current implementation has a lot of limitations, but we think most of them could be easily overcome.

It is important to notice that the 5.5 hours is the time it took to the agent to be trained. After the training is performed, the obtained Q matrix can be used to fit the dataset multiple times with minimal effort. When switching to a Deep Reinforcement Learning algorithm, a great portion of the training process performed over one dataset can be extrapolated to others, further reducing the needed amount of time needed to perform the analysis.

### Future work

To address the limitations of using the likelihood as an unique source of reward, it is necessary to develop a new reward function that takes all the involved elements into account with a proper balance. This is: fitness, number of parameters, execution time, complexity, etc. By using this approach there is a shift in control, everything modelers could tweak before now resides within the reward function. For example: by using the Akaike information criterion or the Bayesian one the agent could address for the number of parameters, or by splitting the data into training and evaluation sets and giving the agent the reward only based on the evaluation one the agent would learn how not to overfit the training set.

To generalize the model definition or in the case covariates are needed it would be necessary to use a Deep Reinforcement Learning algorithm because the state-action space would be too large to be explored in a reasonable amount of time. Other benefit of switching to a Deep Reinforcement Learning algorithm is that the we would not have the restriction of discrete state and actions spaces.

## Data Availability

The raw data, logs and source code can be found on our GitHub repository: https://github.com/Siel/Vial
